# A correlation of ineffective erythropoiesis and dysregulated signaling pathways in myelodysplastic syndromes/neoplasms

**DOI:** 10.1186/s40164-025-00664-1

**Published:** 2025-05-14

**Authors:** Junying Wu, Jinqin Liu, Jia Chen, Lin Yang, Fuhui Li, Tiejun Qin, Zefeng Xu, Jing Liu, Jiaxi Zhou, Lihong Shi, Bing Li, Zhijian Xiao

**Affiliations:** 1 State Key Laboratory of Experimental Hematology, National Clinical Research Center for Blood Diseases, Haihe Laboratory of Cell Ecosystem, Institute of Hematology and Blood Diseases Hospital, Chinese Academy of Medical Sciences & Peking Union Medical College, Tianjin, China; 2 MDS and MPN Centre, Institute of Hematology and Blood Diseases Hospital, Chinese Academy of Medical Sciences & Peking Union Medical College, 288 Nanjing Road, Tianjin, 300020 China; 3 Hematologic Pathology Center, Institute of Hematology and Blood Diseases Hospital, Chinese Academy of Medical Sciences & Peking Union Medical College, Tianjin, China

**Keywords:** Myelodysplastic syndrome/neoplasms, Ineffective erythropoiesis, Signaling pathway changes, Ferroptosis

## Abstract

Over 90% of patients with myelodysplastic syndromes/neoplasms (MDS) exhibit anemia at diagnosis, primarily due to ineffective erythropoiesis. This is characterized by abnormal proliferation and differentiation of erythroid cells influenced by signaling pathways including heme synthesis, ferroptosis, senescence and apoptosis. Despite widespread anemia, the specific mechanisms and pathway alterations at different disease stages are not well understood. This study employed the NUP98-HOXD13 (NHD13) transgenic mouse model, which mimicked the erythroid changes observed in MDS patients, to explore these dynamic pathway changes during disease progression. Based on the severity of anemia and changes in mean corpuscular volume (MCV), four time points were selected: 6 weeks (non-anemic), 12 weeks (mild anemia), 16 weeks (obvious anemia) and 20 weeks (severe macrocytic anemia). The findings indicated that a reduction in erythroid-committed progenitors and impaired erythroid maturation were linked to ineffective erythropoiesis. As the disease progressed, signaling pathways dynamically changed. Heme metabolism and ferroptosis pathways were significantly upregulated in the pre-disease and early disease stages, while senescence and cell cycle pathways were activated in the early stage. The prominent roles of apoptosis, pyroptosis and inflammasome signaling pathways were observed in the late stage. Notably, changes in *Gpx4* and *Ncoa4* expression, along with transmission electron microscopy analysis, suggested that ferroptosis played a critical role in the early stage of the disease. To our knowledge, this is the first report of the signaling pathway dynamics associated with ineffective erythropoiesis during the pathogenesis and progression of MDS, highlighting potential targets for therapeutic intervention at various stages of the disease.

**Supplementary Information:**

The online version contains supplementary material available at 10.1186/s40164-025-00664-1.

## Background

Myelodysplastic syndromes/neoplasms (MDS) are a spectrum of heterogeneous hematological malignancies mainly originating from hematopoietic stem and progenitor cells (HSPCs), characterized by ineffective erythropoiesis, bone marrow (BM) dysplasia, cytopenia and a risk of progression to acute myeloid leukemia (AML) [1]. Lower-risk MDS patients are defined as those with the revised International Prognostic Scoring System (IPSS-R) score of ≤ 3.5 points, with the primary clinical manifestation being refractory cytopenia [2, 3]. One of the main causes of anemia is the presence of ineffective erythropoiesis in the BM, which refers to the excessive destruction of erythroid precursors during their maturation [1]. This process involves abnormalities in both the'quality'and'quantity’ of erythroid cells.

Recent research [4] has shown that as colony-forming unit-erythroid (CFU-E) cells transition into proerythroblasts, those that upregulate transferrin receptors (CD71), exhibit vigorous heme synthesis are more prone to reactive oxygen species (ROS)-mediated damage. This indicates that excessive heme synthesis is closely linked to ineffective erythropoiesis. Robust heme synthesis results in elevated intracellular iron levels, leading to increased production of ROS, ultimately mediating ferroptosis [4–7].

In addition to the heme synthesis and ferroptosis pathways, several other signaling pathways have been implicated in the ineffective erythropoiesis in MDS [8–12]. Maratheftis et al. [9] found increased apoptosis within CD34^+^ cells from MDS patients, contributing to ineffective hematopoiesis. Emerging research has shown that the Nod-like receptor 3 (NLRP3) inflammasome was activated, driving caspase-1-dependent pyroptosis in MDS [13–16]. In our previous work [12], premature hemoglobin production and early senescence markers were observed in MDS patients, suggesting the concepts of precocity and premature aging related to ineffective erythropoiesis. Therefore, multiple pathways are involved in ineffective erythropoiesis. However, their dynamic changes in MDS remain unclear.

In this study, the NUP98-HOXD13 (NHD13) transgenic mice model was utilized to delineate pathway alterations from preclinical to advanced stages of MDS. Heme metabolism and ferroptosis pathways were notably upregulated in preclinical and early stages. Cellular senescence and cell cycle pathways were activated during the early stage, while the roles of apoptosis, pyroptosis and inflammasome pathways became prominent in the late stage.

## Methods

### Patients

A total of 1208 newly diagnosed primary MDS patients between August, 2016 and June, 2023 in our center were included in the study. Retrospective complete blood counts (CBC) before diagnosis were collected from all patients, and 783 of them had traceable CBC information prior to diagnosis. Written informed consent was obtained from all patients in accordance with the Declaration of Helsinki. The study was approved by the Ethics Committee of the Blood Disease hospital, Chinese Academy of Medical Sciences & Peking Union Medical College.

### Gene expressions in MDS patient’s database

Gene expression data were obtained from the GEO database, including 183 MDS CD34^+^ samples and 17 controls (GSE19429), along with data for 55 MDS patients and 11 healthy controls (GSE4619), as previously described [17, 18].

### Mice

C57BL/6 (WT) and C57BL/6-Tg (Vav1-NUP98/HOXD13) G2 Apla/J (NHD13) mice [19] were purchased from Jackson Laboratory and all mice were of a pure C57BL/6 genetic background. All animal studies were approved by the Institutional Animal Care and Use Committee of State Key Laboratory of Experimental Hematology.

### Isolation of murine BM nucleated cells

Murine BM nucleated cells (BMNCs) were harvested followed as previous described [20]. Murine BMNCs were enriched for c-kit positive cells using CD117 MicroBeads (Miltenyi), followed by separation with an AutoMACS Pro separator (Miltenyi). BM c-kit^+^ cells were utilized for cell sorting, during which we specifically isolated erythroid-committed progenitor (ErP, Lineage^−^c-kit^+^Sca1^−^ CD34^−^Fcγ^−^CD71^+^) cells using BD FACS Aria III flow cytometer (BD Biosciences) as described previously [21].

To assess the cellular composition of the sorted ErP population, we employed a published flow cytometry strategy described by *Pronk et al* [22]. While temporal variations in CFU-E and pre-CFU-E proportions were observed within ErP populations, age-matched WT and NHD13 cohorts maintained comparable frequencies across different time points (Fig S1). Although this sorting strategy is well-established in steady-state hematopoiesis, functional confirmation was precluded by limited erythroid cell yields in severely anemic NHD13 bone marrow. Thus, the flow cytometry-purified ErP populations may not represent functionally equivalent cell populations across all experimental conditions.

### Flow cytometric analysis

Murine BMNCs were isolated following the method mentioned above. Spleen nucleated cells (SPLNCs) were processed by homogenizing and filtering through a 70-μm strainer (BD Biosciences). Peripheral blood (PB) was collected from the retro-orbital vein. Single-cell suspensions from BM, spleen and PB were stained with specific antibodies in 2% FBS/PBS for 30 min on ice after RBC lysis. Erythroid precursors were stained with specific antibodies in 2% FBS/PBS for 30 min on ice. RBC lysis was not performed on the erythroid precursor samples to avoid loss of terminal stage precursors. Detailed information can be found in Supplemental Materials.

Flow cytometric analysis of terminal erythroid differentiation was followed as outlined by Liu et al*.* [23], Morphology of sorted cells was confirmed by H&E staining (Fig S2). BM and spleen cells were incubated with anti-mouse TER119 APC (BD Biosciences) and CD44-PE (BioLegend) to identify progressive stages of erythroblast differentiation, as depicted in Fig S2. The classification of these stages was achieved using a gating strategy that involved TER119, CD44 and forward scatter. The assessment of erythroid differentiation involved quantifying the proportion of each stage of erythropoiesis relative to the total erythroblasts in each BM sample. Subsequent analyses were conducted on a FACS Canto II flow cytometer (BD Biosciences) and data were processed using FlowJo software version 10 (Tree Star Inc.).

### RNA-Seq and bioinformatics

ErPs from NHD13 transgenic mice and age-matched WT mice were sorted at different time points corresponding to the severity of anemia (6, 12, 16 and 20 weeks). Transcriptome sequencing and bioinformatics analysis were then performed. The processes of RNA amplification, library preparation, and data preprocessing were carried out by Novogene Co. (Beijing, China).

Each group at each time point included 5 mice, ensuring sufficient independent biological replicates. From these, 4–5 samples with similar transcriptional profiles, based on principal component analysis (PCA), were selected for subsequent bioinformatics analysis. PCA plots showed high consistency among samples within each group, indicating minimal variability between experimental replicates. Batch effect correction was applied, and joint analysis of the selected samples was performed (Fig S3). The differential genes at each time point between NHD13 and age-matched WT mice (log^2^fold change > 1, adjusted *P-*value < 0.05) were first screened and a union of these genes was formed. All differential genes in the union were then subjected to *K-means* clustering using the ClusterGVis package, grouping genes into clusters with similar expression patterns to identify gene expression profiles across different biological states. Subsequently, GSVA and GSEAbase were employed to perform gene set enrichment analysis, calculating the scores of each sample in custom pathway gene sets to evaluate the activity levels of these pathways in the samples.

### In vitro colony-forming assays

BMNCs at 2 × 10^4^ per well and SPLNCs at 1 × 10^5^ per well were seeded in triplicate using methylcellulose medium (Methocult M3434, Stem Cell Technologies). The colonies of burst-forming unit-erythroid (BFU-E), granulocyte–macrophage colony-forming unit (CFU-GM), and colony-forming unit-granulocyte, erythrocyte, macrophage, and megakaryocyte (CFU-GEMM) were counted on the seventh day of incubation at 37℃ in 5% CO_2_. 4 × 10^5^ BMNCs or 8 × 10^5^ SPLNCs were plated in methylcellulose colony assay medium (MethoCult GF M3334, Stem Cell Technologies). CFU-E were counted after 48 h of incubation at 37℃ in 5% CO_2_.

### Preparation and staining of bone marrow smears

Cytospin BM smears were prepared by suspending the murine BMNCs at a density of 1 × 10^6^ cells/mL, followed by centrifugation at 600 rpm at room temperature for 5 min onto glass slides. The slides were then subjected to Periodic Acid-Schiff (PAS) staining (G1281, Solarbio) and Prussian blue iron staining (G1422, Solarbio) according to the manufacturer’s instructions.

### Serum erythropoietin and iron metabolism markers measurement

Serum mouse erythropoietin (E-EL-M3058, Elabscience) was measured by enzyme-linked immunosorbent assay (ELISA) according to the manufacturer’s instructions. Serum hepcidin was measured using a Mouse Hepc (Hepcidin) ELISA kit (Elabscience). Integra 800 Automated Clinical Analyzer was used to measure serum iron; serum transferrin (E-EL-H6028) and ferritin (E-EL-M0491) concentration were measured according to the manufacturer’s protocols (Elabscience).

### Electron microscopy

Murine BM clumps were prepared transition electron microscopy (TEM) and scanning electron microscopy according to standard procedures, detailed in Supplemental Methods.

### Statistical analysis

Data are presented as the mean ± standard error of the mean (SEM). Statistical analysis was conducted using an unpaired two-tailed t-test. Survival comparisons were assessed with Kaplan–Meier survival analysis and the log-rank test. All statistical analyses were performed using GraphPad Prism software (San Diego, CA). A *P*-value of less than 0.05 was considered to indicate statistical significance.

## Results

### Decreased hemoglobin and increased mean corpuscular volume are found in patients with MDS before diagnosis

We performed a retrospective analysis of all newly diagnosed primary MDS patients from 2016 to 2023 at our center. Among them, 783 subjects had pre-diagnosed blood counts. Clinical and hematological features of these patients at diagnosis were reported in Table S1.

To explore pre-diagnostic changes in mean corpuscular volume (MCV) and hemoglobin (HGB) levels, we analyzed comprehensive blood count data from these 783 subjects, with the earliest data extending up to 20 years prior to diagnosis. Linear regression showed a gradual HGB decline (− 2.78 g/L/year) and an MCV increase (+ 0.83 fL/year) (Fig S4), suggesting prolonged ineffective hematopoiesis in MDS patients before diagnosis.

### NHD13 mice similarly exhibit a gradual increase in MCV and a decrease in HGB, suitable as a model for lower-risk MDS

**Fig. 1:**
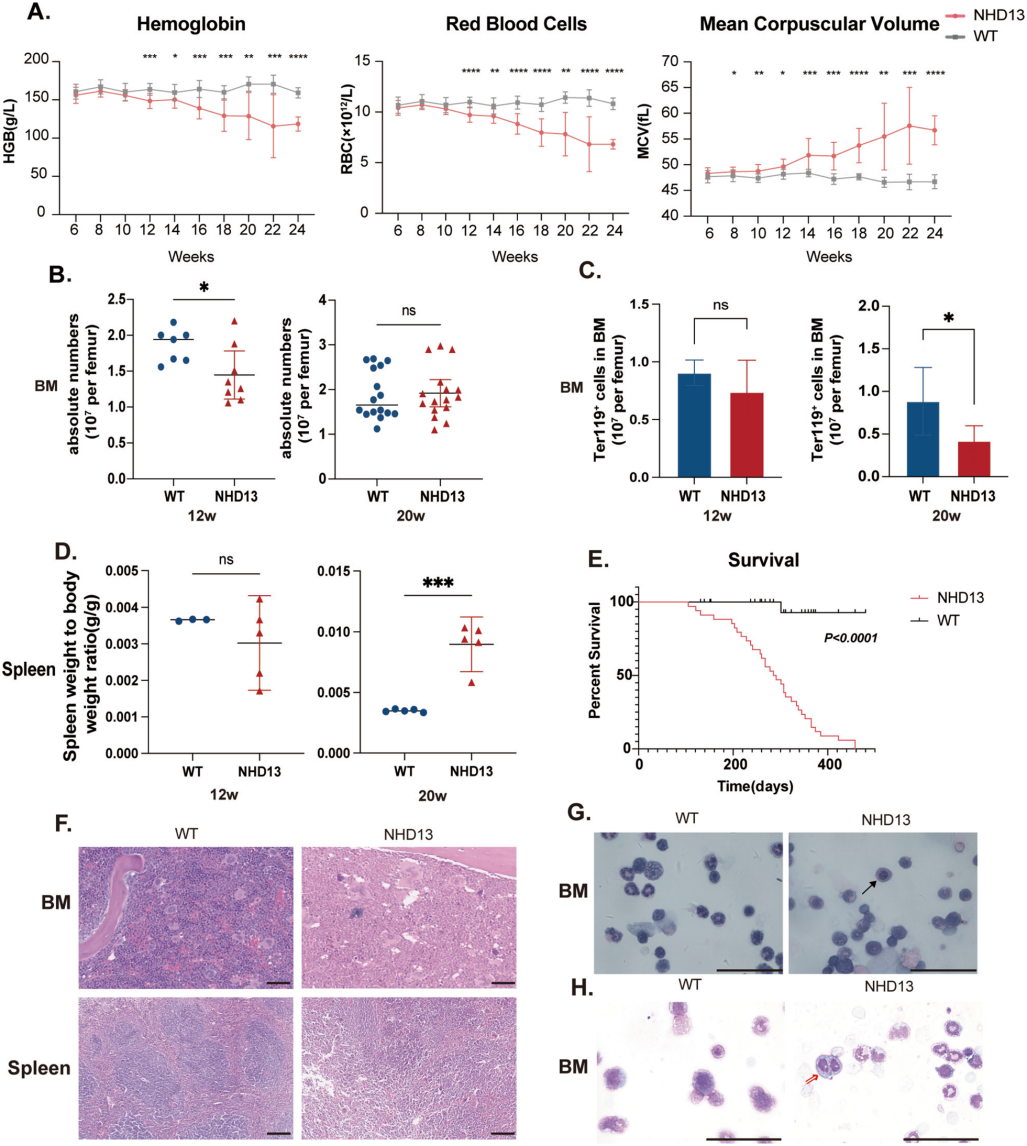
NHD13 mice exhibit increased MCV and decreased HGB, indicating increased ineffective erythropoiesis. **A** Hemoglobin (HGB), red blood cell and mean corpuscular volume (MCV) in peripheral blood (PB) of different time points of NHD13 mice and WT mice (n = 12–20 mice per group). **B**–**D** Comparison of disease phenotypes between NHD13 mice and WT mice at 12 and 20 weeks. **B** Absolute BM cell numbers per single femur at 12 and 20 weeks of age. **C** Absolute Ter119^+^ erythroid cells per femur at 12 and 20 weeks of age (n = 3–5 mice per group). **D** Spleen weight relative to body weight between NHD13 and WT mice at 12 and 20 weeks (n = 3–5 mice per group). **E** Kaplan–Meier survival analysis of the NHD13 and WT mice (NHD13, n = 34; WT, n = 30). **F** Morphology of BM and spleen biopsy specimens from 20-week NHD13 and age-matched WT mice. BM and spleen histopathological sections stained with hematoxylin and eosin (H&E). NHD13 mice showed significant erythroid hypoplasia in BM specimens and disrupted structure of the spleens, characterized by increased red pulp, scale bar: 200μm. **G** The periodic acid-Schiff (PAS) staining images of cytospin nucleated erythrocytes from BM of mice with different genotypes highlight PAS-positive nucleated erythrocytes in NHD13 mice; → : PAS-positive nucleated erythrocyte. **H** Wright-Giemsa staining on cytospin BM smears from NHD13 mice and WT mice; ⇒: binucleate erythrocyte; Panel G-H, scale bar: 50μm

To model human MDS, we utilized NHD13 transgenic mice due to limited access to pre-diagnostic MDS patient samples. Continuous CBC monitoring in NHD13 and age-matched WT mice revealed that, no significant differences in MCV or HGB were seen at 6 weeks, but MCV rose after 8 weeks and HGB declined after 12 weeks in NHD13 mice, mirroring ineffective erythropoiesis in MDS patients (Fig. 1A).

Therefore, we selected four time points in NHD13 mice to represent MDS progression stages: 6 weeks (no anemia), 12 weeks (mild anemia), 16 weeks (obvious anemia), and 20 weeks (severe macrocytic anemia). These time points corresponded to the pre-disease, early, middle and late stages of patients with MDS.

The evolution of the disease phenotype in NHD13 mice was compared to age-matched WT mice, showing that BM cellularity was comparable or slightly reduced in NHD13 mice (Fig. 1B**, **Fig S5 F). While liver and spleen weights were similar until 16 weeks, by 20 weeks, NHD13 mice showed enlarged livers and spleens, indicating intensified extramedullary hematopoiesis as HGB declined (Fig. 1D, Fig S5B–D). Additionally, NHD13 mice had a shorter lifespan compared to WT mice, with the majority of NHD13 mice succumbing to disease-related complications by 40 weeks, further supporting the progressive nature of MDS in this model (Fig. 1E, Table S3).

Thus, the NHD13 mouse model effectively replicates the key features and progression of ineffective erythropoiesis in MDS, supporting its suitability for related studies.

### Erythroid dysplasia, progenitor cell exhaustion and impaired terminal erythropoiesis are observed in NHD13 mice

We first examined BM and spleen histology in 20-week-old NHD13 and WT mice. NHD13 mice displayed significant erythroid hypoplasia in BM and compensatory erythropoiesis in spleens (Fig. 1F). Cytospin BM smears with Wright-Giemsa and PAS staining revealed marked erythroid dysplasia, such as binucleated erythrocytes (Fig. 1G–H).

**Fig. 2:**
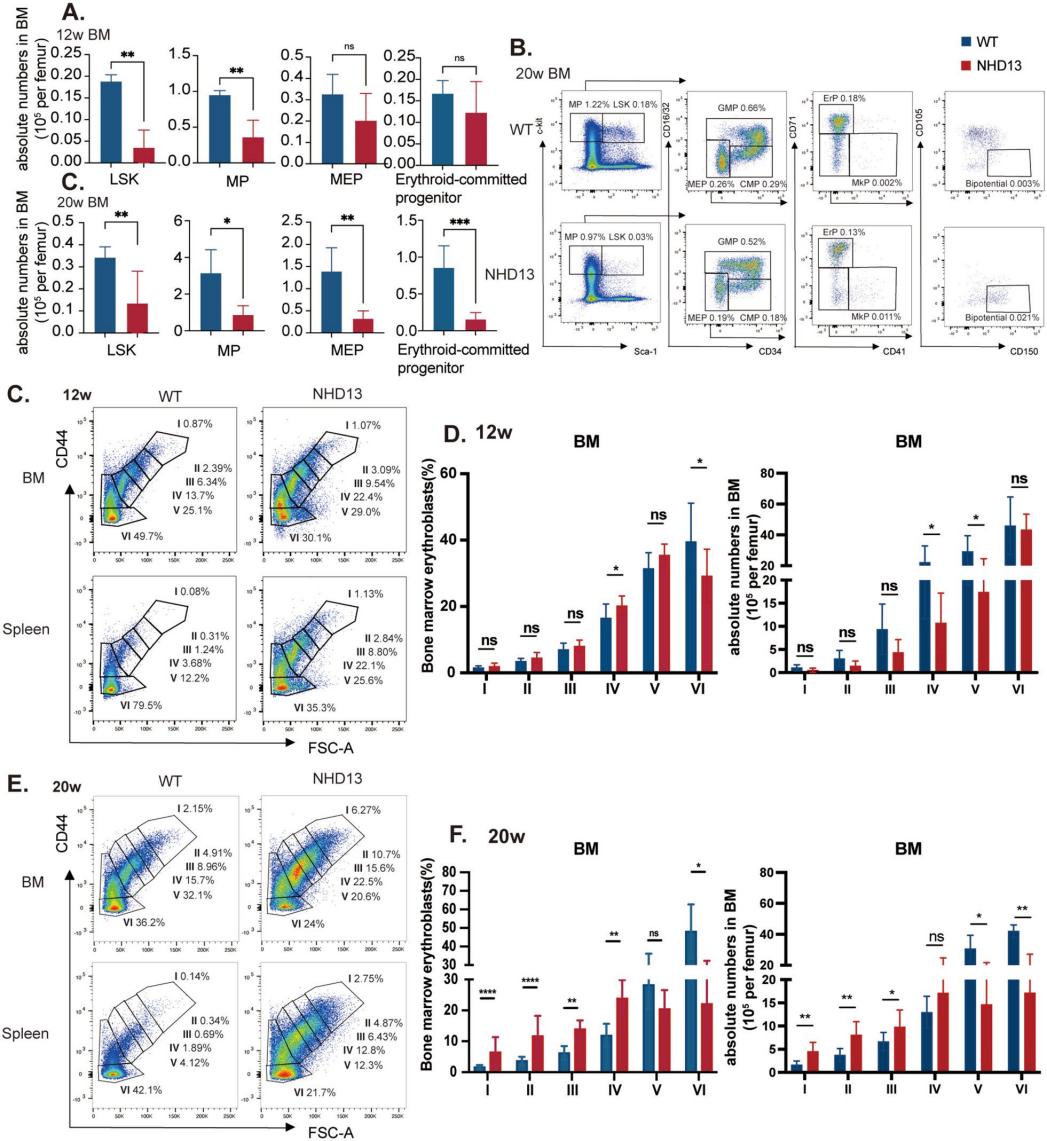
Erythroid progenitor cell exhaustion and impaired terminal erythropoiesis are observed in NHD13 mice. Quantification of hematopoietic progenitor cell populations (**A**) and representative flow cytometric plots (**B**) in the BM of NHD13 and WT mice at 12 and 20 weeks (n = 5–6 per group). Absolute cell numbers were normalized to a single femur. **C**–**F** Comparison of the proportions and absolute cell counts (per femur) of erythroblasts at different stages of terminal erythroid differentiation in the BM of NHD13 and WT mice at 12 (**C**, **D**) and 20 weeks (**E**, **F**) of age. CD44 levels and forward scatter (FSC) were analysed to define various developmental stages of erythroblasts (n = 8–9 per group). All the data are shown as mean ± s.e.m. A two-tailed unpaired Student t test was performed between means of two groups. ns, not significant, *P < 0.05, **P < 0.01, ***P < 0.001, ****P < 0.0001. LSK: Lin^−^Sca-1^+^c-kit^+^ cells; MP: multipotent progenitors; GMP: granulocyte/macrophage progenitors; CMP: common myeloid progenitors; MEP: megakyocyte-erythroid progenitors; ErP: erythroid-committed progenitors (Lin^−^ckit^+^Sca-1^−^CD34^−^Fcγ^−^CD71^+^); MkP: Megakaryocyte-committed progenitors; Bipotential: bipotential progenitors. I: proerythroblasts; II: basophilic erythroblasts; III: polychromatic erythroblasts; IV: orthochromatic erythroblasts; V:reticulocytes; VI: mature red blood cells

To further investigate the causes of anemia progression, we conducted flow cytometry analysis of HSPCs and erythroid differentiation in NHD13 and WT mice at four time points. At 6 weeks, NHD13 mice had similar levels of multipotent progenitor (MP, Lin^−^Sca-1^−^c-kit^+^) cells, megakaryocyte/erythroid progenitor (MEP) cells and ErP cells compared to WT controls (Fig S6 A, B). After 16 weeks, there was a slight reduction in MEPs and ErPs (Fig S6B), which became more pronounced by 20 weeks (Fig. 2A, B). In contrast, at 20 weeks, proportions of MEPs and ErPs were significantly elevated in the spleens of NHD13 mice, reflecting compensatory splenic erythropoiesis as anemia advanced (Fig S6 C, D).

Further analysis of erythroid terminal differentiation using CD44, and forward scatter showed a slight reduction in the proportion of mature erythrocytes in NHD13 BM at 12 weeks, with increased nucleated erythroblasts in the spleen at 12 weeks (Fig. 2C, D, Fig S7). At 20 weeks, there were substantial shifts in terminal erythroid maturation, with a rise in primitive nucleated erythroblasts and fewer mature erythrocytes (Fig. 2E, F). Additionally, colony formation assays confirmed HSPC dysfunction in NHD13 mice, showing reduced CFUs in BM (Fig S8).

Taken together, our data suggest that ineffective erythropoiesis in NHD13 mice involves erythroid dysplasia, exhaustion of HSPCs and blocked terminal differentiation, with compensatory splenic erythropoiesis mitigating these effects.

### Stage-specific signaling pathway activation with worsening ineffective erythropoiesis in MDS

**Fig. 3:**
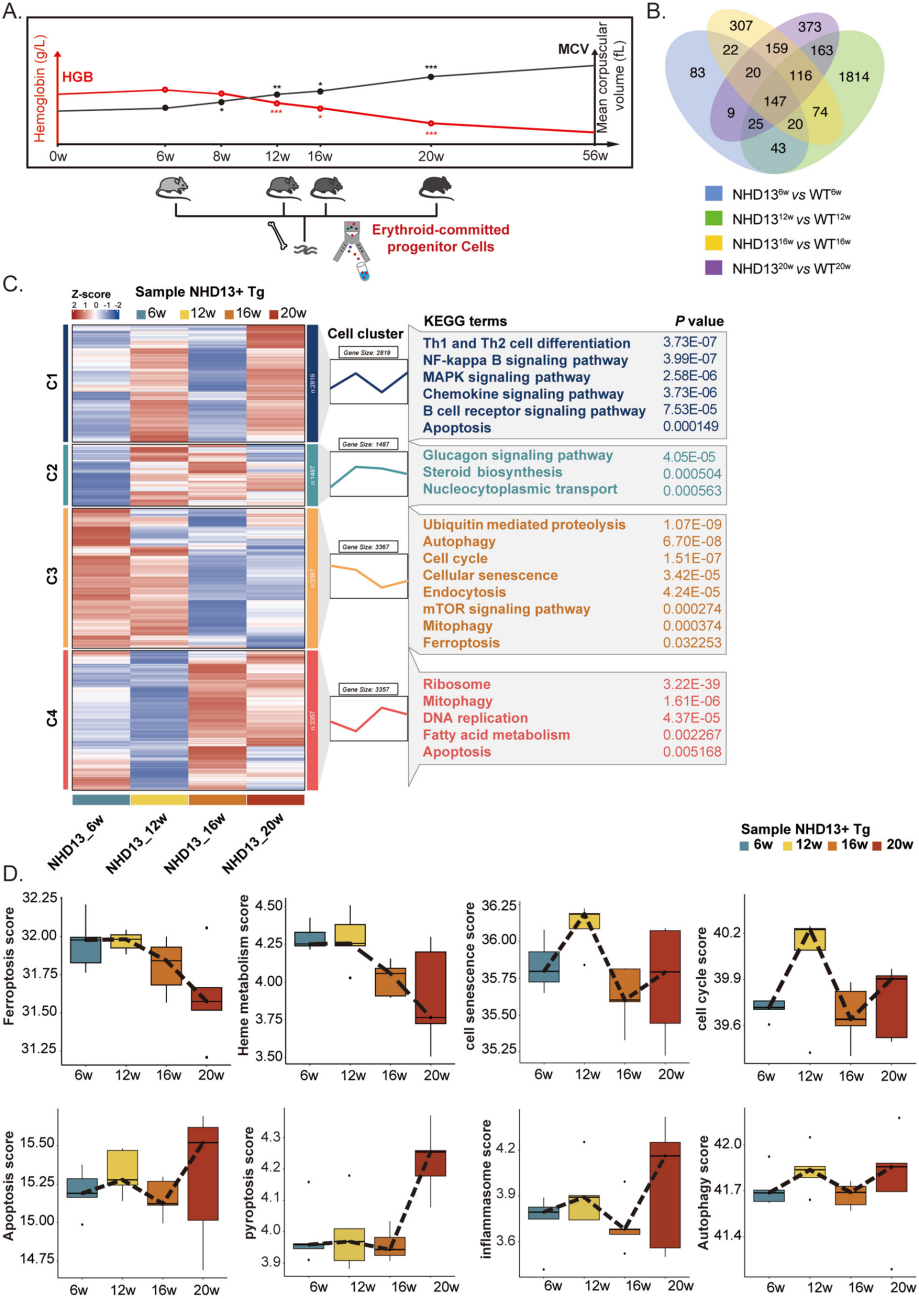
Gene expression and pathways changes in erythroid-committed progenitors of NHD13 mice at various time points. **A** Erythroid-committed progenitors (ErPs) were sorted from mice of different genotypes at various time points, and RNA was extracted for transcriptome sequencing analysis. **B** A Venn diagram identifies genes that are significantly differentially expressed in ErPs from different time points in NHD13 mice, in comparison with their respective controls. The numbers of overlapping genes are also indicated. **C** Gene expression profiles of erythroid-committed progenitors from NHD13 mice at various time points were clustered into different gene clusters, with each cluster subjected to KEGG enrichment analysis. **D** Gene set scores for various signaling pathways in ErPs from NHD13 mice at different time points

To further delineate the potential mechanisms underlying signaling pathway alterations associated with the exacerbation of ineffective erythropoiesis in this MDS mouse model, RNA sequencing was performed in ErPs from NHD13 mice and age-matched WT mice at four time points selected above (Fig. 3A).

We initially identified the differentially expressed genes (log^2^fold change > 1, adjust *P* < 0.05) between NHD13 and age-matched WT mice at different time points and generated a union set of 3375 differentially expressed genes (Fig. 3B). Subsequently, by performing clustering analysis on the expression levels of these genes, genes with similar expression patterns were grouped together. The distinct gene expression patterns reflected the functional changes of ErPs at different time points.

All differentially expressed genes can be classified into four distinct clusters based on their expression patterns. Kyoto Encyclopedia of Genes and Genomes (KEGG) enrichment analysis on these four clusters were performed (Fig. 3C). Cluster 1 genes were highly expressed at 12 and 20 weeks, which were mainly enriched in MAPK and NF-κB signaling pathways. Cluster 2 genes expression was increased at 12 week and decreased thereafter, including glucagon and steroid synthesis pathways. Cluster 3 genes peaked in expression at 6 weeks and progressively decrease with disease progression, enriching pathways including autophagy, cellular senescence, cell cycle and ferroptosis pathways. Cluster 4 genes showed a significant increase in expression at 16 weeks, followed by a stable expression pattern, and were enriched in pathways related to ribosome, mitophagy, DNA replication, lipid metabolism and apoptosis. Pathways related to ineffective erythropoiesis such as apoptosis, autophagy, cell cycle and aging were enriched in more than one gene cluster, indicating that different genes within these pathways exhibit distinct expression patterns.

Previous data have shown that potential mechanisms of ineffective erythropoiesis in MDS may include aberrant activation of pathways related to heme metabolism, cellular senescence, apoptosis, inflammasomes and ferroptosis pathways [4, 8–12]. To assess the overall trends in signaling pathways associated with the exacerbation of ineffective erythropoiesis, we aggregate all genes within a specific pathway and calculate the total score of this gene set at various time points (Fig. 3D). Heme metabolism [4, 24] and ferroptosis signaling pathways were highly expressed in the pre-disease and early disease stage, but their expression levels decreased during the disease progression. Pathways related to cellular senescence and cell cycle were significantly upregulated during early stage but decreased in the later phase. In contrast, apoptosis, pyroptosis and inflammasomes pathways were abnormally activated in the late stage of the disease. Autophagy pathway was stably expressed throughout the disease.

These data suggested that multiple pathways contribute to ineffective erythropoiesis at different stages of the disease, with early involvement of the ferroptosis signaling pathway.

### Increased ferroptosis gene expressions and related morphological changes in early-stage MDS erythroid progenitor cells

As mentioned before, ferroptosis pathway was significantly upregulated in both preclinical and early disease stages but downregulated in the late stage. Ferroptosis was uniquely triggered by dysregulated cellular iron levels and lipid hydroperoxides [5]. Therefore, we further evaluate the iron metabolism and the activation of ferroptosis pathway in NHD13 mice compared to WT mice during the early and late stage of the disease (at 12 and 20 weeks).

**Fig. 4:**
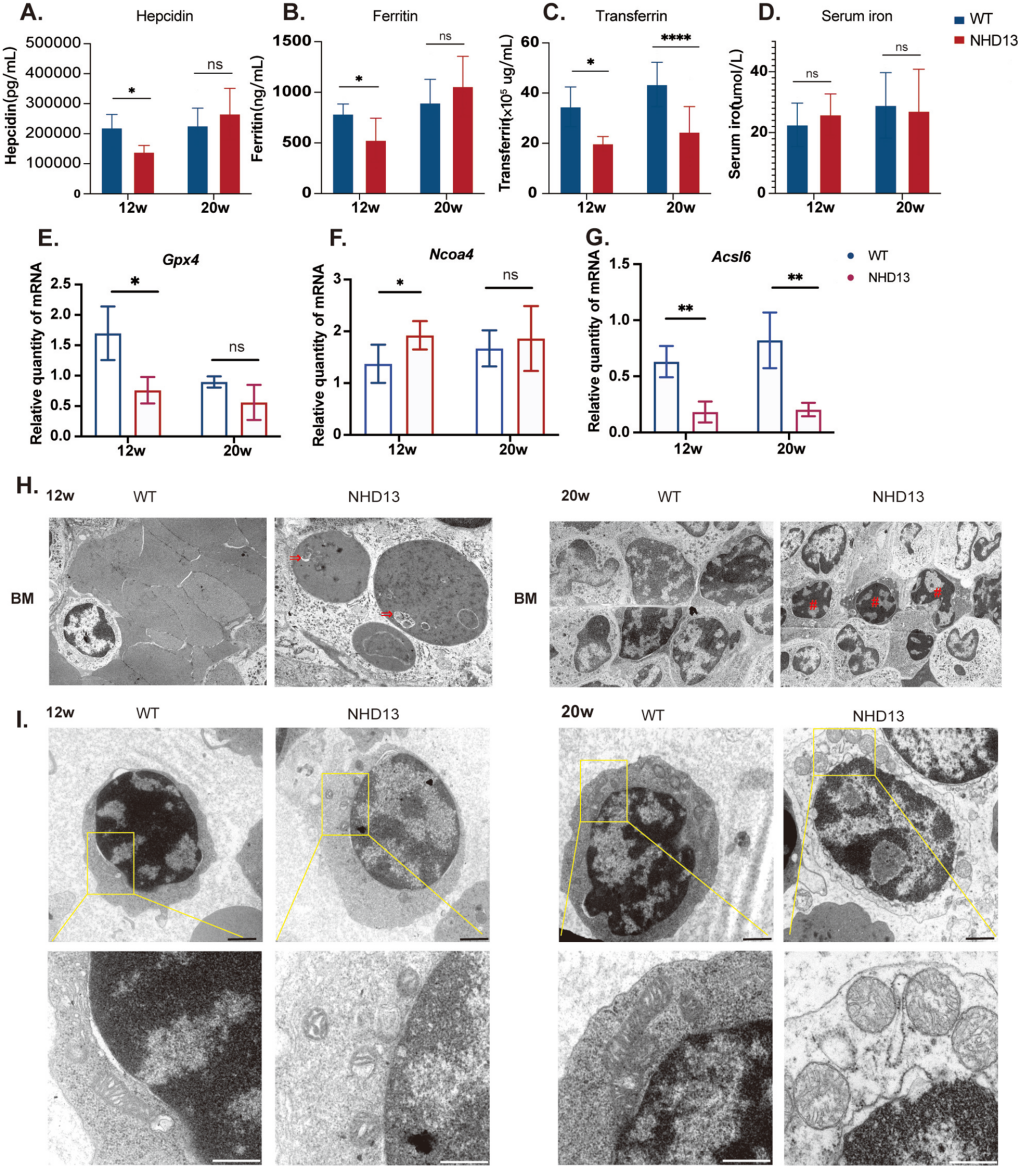
Imbalanced iron metabolism, increased ferroptosis gene expressions and morphological changes in early-stage MDS erythroid progenitors. **A**–**D** NHD13 mice exhibit dysregulation of iron metabolism markers in early stage of the disease (n = 10–12 per group). **E**–**G** Observations reveal changes in the expression of multiple ferroptosis-related genes (n = 3–5 per group). **H** Ultrastructural analysis of erythroid cells in BM from NHD13 and WT mice at 12 and 20 weeks, imaged at 3000 × for BM via electron microscopy. ⇒: Mitophagy; #: Abnormal nuclear condensation and apoptosis in mid to late erythroblasts. **I** Representative transmission electron microscopy images comparing mitochondrial ultrastructure in erythroid cells from the BM of 12-week and 20-week-old NHD13 and WT mice (upper plots: scale bar, 2 μm; lower plots: scale bar, 500 nm). All the data are shown as mean ± s.e.m. ns, not significant, *P < 0.05, **P < 0.01, ***P < 0.001, ****P < 0.0001

Previous studies indicated that hepcidin, a master regulator of iron metabolism, contributed to iron overload in MDS patients [25]. Ferritin is essential for iron storage, protecting cells from oxidative damage by sequestering excess iron [26]. Transferrin is central to iron trafficking [25]. Our results consistently showed elevated serum erythropoietin (EPO) and low serum hepcidin in NHD13 mice compared to WT mice at 12 weeks (Fig S9 A, Fig. 4A). Additionally, serum ferritin and transferrin were reduced in NHD13 mice, while serum iron levels remained comparable between the two groups (Fig. 4B–D). At 20 weeks, EPO remained elevated and transferrin levels were low in NHD13 mice, but ferritin, hepcidin and serum iron levels showed no significant differences compared to WT mice (Fig. 4A–D, Fig S9 A). Furthermore, Perl’s Prussian blue staining confirmed increased BM iron deposition in NHD13 mice (Fig S9B). Thus, these findings indicated that insufficient serum hepcidin, ferritin and transferrin levels at early disease stage may contribute to iron overload. Dysregulation of iron metabolism further exacerbates lipid peroxidation and enhances oxidative stress damage to cells.

Next, we isolated BM erythroid progenitor cells from 12-week and 20-week NHD13 mice as well as age-matched WT mice. In line with the activation of the ferroptosis pathway, changes in the expression of several ferroptosis-related genes were observed.

In previous investigations, glutathione peroxidase 4 (*Gpx4*) is considered as a crucial regulator in preventing ferroptosis [27, 28], while nuclear receptor coactivator 4 (*Ncoa4*) mediates the autophagic degradation of ferritin, both of which are essential for maintaining iron homeostasis [29, 30]. Concurring with the alterations in the ferroptosis pathway described above, the expression of *Gpx4* decreased while that of *Ncoa4* increased during the early stage of the disease (Fig. 4E, F). In contrast, during the late stage of the disease, the expression levels of *Gpx4* and *Ncoa4* did not exhibit notable changes. Acyl-CoA synthetase long-chain family member 6 (*Acsl6*) is primarily involved in lipid synthesis and metabolism, which are key components of the lipid metabolic pathways and the deregulation of lipid metabolism undoubtedly influence cellular sensitivity to ferroptosis [31, 32]. Compared to WT mice, the expression of *Acsl6* in NHD13 mice was reduced throughout all stages of the disease (Fig. 4G).

In addition, gene expression microarray analysis of primary BM CD34 + cells from 183 MDS patients and 17 healthy controls (GSE19429), and 55 MDS patients and 11 healthy controls (GSE4619), revealed elevated *NCOA4* expression in the RA and RAEB1 subtypes of MDS compared to healthy controls (Fig S10 A). Further validation by quantitative polymerase chain reaction (qPCR) in BMNCs from MDS patients confirmed higher *NCOA4* expression in lower-risk MDS patients (Fig S10B).

Furthermore, ferroptosis is uniquely characterized by distinct morphological and biological features [33–35]. Compared to WT mice, TEM analysis revealed that in 12-week NHD13 mice with mild anemia, erythroid cells in the BM displayed ferroptosis-specific changes of mitochondria, including mitochondrial swelling and enhanced membrane density compared to age-matched WT mice (Fig. 4H, I).

In 20-week NHD13 mice with severe anemia, enlarged mitochondria and mitochondrial autophagy were observed in the BM (Fig. 4H, I). Compared to WT mice, 20-week NHD13 mice showed erythroblast nuclear chromatin clumping, nuclear envelope disruption, and enlarged nuclear pores in the BM, with observable increased apoptosis of erythroid precursor cells (Fig. 4H).

Taken together, our results suggested dynamic changes in signaling pathways related to ineffective erythropoiesis during MDS progression, including early activation of the ferroptosis pathway.

## Discussion

Anemia due to ineffective erythropoiesis is the most prevalent clinical manifestation of MDS, with over 90% of patients experiencing varying degrees of anemia at diagnosis, which often follows a prolonged latency period prior to the onset of anemia [36, 37]. Although several signaling pathways related to ineffective erythropoiesis have been identified, their changes from preclinical to severe symptomatic stages of MDS remain unclear [12–16, 38]. This study investigated pathway dynamics related to ineffective erythropoiesis by utilizing a MDS mouse model at different disease stages and analyzing ErPs transcriptome sequencing data, highlighting the potential for stage-specific treatments.

In MDS patients, MCV is frequently found to be abnormally elevated, particularly in cases where BM blasts are less than 5% [39]. This macrocytic anemia in MDS is often due to an imbalance between heme and globin production, indicating ineffective erythropoiesis [40, 41]. A retrospective CBC analysis of primary MDS patients revealed a gradual MCV increase and steady HGB decline 2–3 years preceding diagnosis, aligning with a previous study [42]. Similarly, NHD13 transgenic mice displayed MDS-like erythrocyte changes: no significant differences in HGB and MCV at 6 weeks compared to WT mice, a decrease in MCV by 8 weeks, and a gradual HGB decline from 12 weeks onward. This MDS model also replicated key MDS features, including HSPC failure, morphological dysplasia and excessive apoptosis, supporting its use for investigating signaling pathway alterations associated with ineffective erythropoiesis.

To understand the dynamic changes in pathways associated with ineffective erythropoiesis, ErPs were isolated from NHD13 mice at 6, 12, 16, and 20 weeks, representing stages from preclinical to advanced disease. As ineffective erythropoiesis progressed, genes within specific pathways exhibited distinct patterns of expression variation. Gene set scoring allowed for a precise assessment of overall trends within these pathways.

Consistent with the findings of Doty et al. [4], marked upregulation of the heme metabolism pathway was evident in the early-stage disease, leading to robust heme synthesis, cell enlargement, ROS accumulation and the activation of apoptosis and ferroptosis [4]. These processes collectively contribute to excessive erythrocyte destruction [40, 41]. Heme synthesis pathway inhibitors, like succinylacetone, can suppress heme production, thereby mitigating damage from excessive heme and ROS, decreasing cell mortality and enhancing red blood cell maturation [4].

The activation of the heme synthesis pathway promotes an influx of iron into cells and iron accumulation [4]. The increase in intracellular redox-active iron (Fe^3^⁺) catalyzes ROS production via Fenton and Haber–Weiss reactions, overwhelming antioxidant defenses, disrupting lipid membranes through lipid peroxidation, ultimately inducing ferroptosis [5–7]. Therefore, the ferroptosis and the heme metabolism pathways exhibited the same trend of early activation in our result.

Based on these findings, comparisons of iron metabolism indices and ferroptosis-related genes between early and later disease stages in MDS and WT mice substantiated the early activation of ferroptosis pathway. In early-stage NHD13 mice, iron metabolism dysregulation was observed. Systemic hypoxia may stimulate erythropoietin (EPO) production [43, 44]. This, in turn, enhances the production of growth differentiation factor 15 and erythroferrone in erythroblasts, effectively suppressing hepcidin synthesis and thereby regulating iron levels [45–49]. Decreased hepcidin levels in MDS mice prompt compensatory iron absorption from the intestine and enhance release from macrophage stores, resulting in systemic iron overload [44, 50–52]. Meanwhile, iron accumulation in tissues accounts for the similar serum iron levels in NHD13 and WT mice. Concurrently, an inefficiency in iron utilization contributes to diminished iron transport, reflected by reduced transferrin levels, aligning with findings from prior research [50].

Apart from iron homeostasis imbalance, our study also corroborated aberrant expression of ferroptosis-related genes in the early stages of the disease, alongside the previously alterations in the ferroptosis pathway. *Gpx4*, pivotal in preventing ferroptosis, exhibited reduced expression, while *Ncoa4*, responsible for the autophagic degradation of ferritin, showed elevated levels in ErPs from NHD13 mice in the early stages [5–7]. Moreover, ferroptosis-specific mitochondrial changes have been observed by TEM. These findings further confirmed that abnormal activations of the ferroptosis pathway in the initial stages of disease in NHD13 mice.

Therefore, considering the detected aberrant activation of the ferroptosis pathway during the early stages of the disease, early detection of iron metabolism markers in patients could indicate the presence of ineffective erythropoiesis. Potential prevention of ferroptosis and deceleration of disease progression could be achieved by early regulation of iron metabolism or enhancement of cellular antioxidant capacity, through the use of iron chelation therapy, antioxidants and cell membrane stabilizers [50, 53, 54].

Subsequently, we discovered that in the early stages of the disease, there is an upregulation of pathways related to cellular aging and cell cycle. Previous studies [55, 56] have demonstrated that MDS patients exhibit shorter telomeres in hematopoietic cells compared to healthy individuals. Huang et al*.* [12] also found that erythroid progenitors from MDS patients prematurely produce hemoglobin during differentiation, with an upregulation of aging-related pathway. Additionally, iron metabolism disorders are associated with telomere shortening, as reported in recent studies [57]. These results reveal that an imbalance in iron homeostasis not only leads to increased intracellular ROS, implicated in causing DNA damage and inducing cellular aging, but also prompts the body to accelerate hemoglobin production to compensate for ineffective erythropoiesis through premature cellular development.

In the late stage of the disease, abnormally high expression of pathways involved in apoptosis, pyroptosis and inflammasome activation was observed in erythroid progenitors, indicating that these pathways contribute to erythroid cell damage and exacerbated BM failure.

**Fig. 5:**
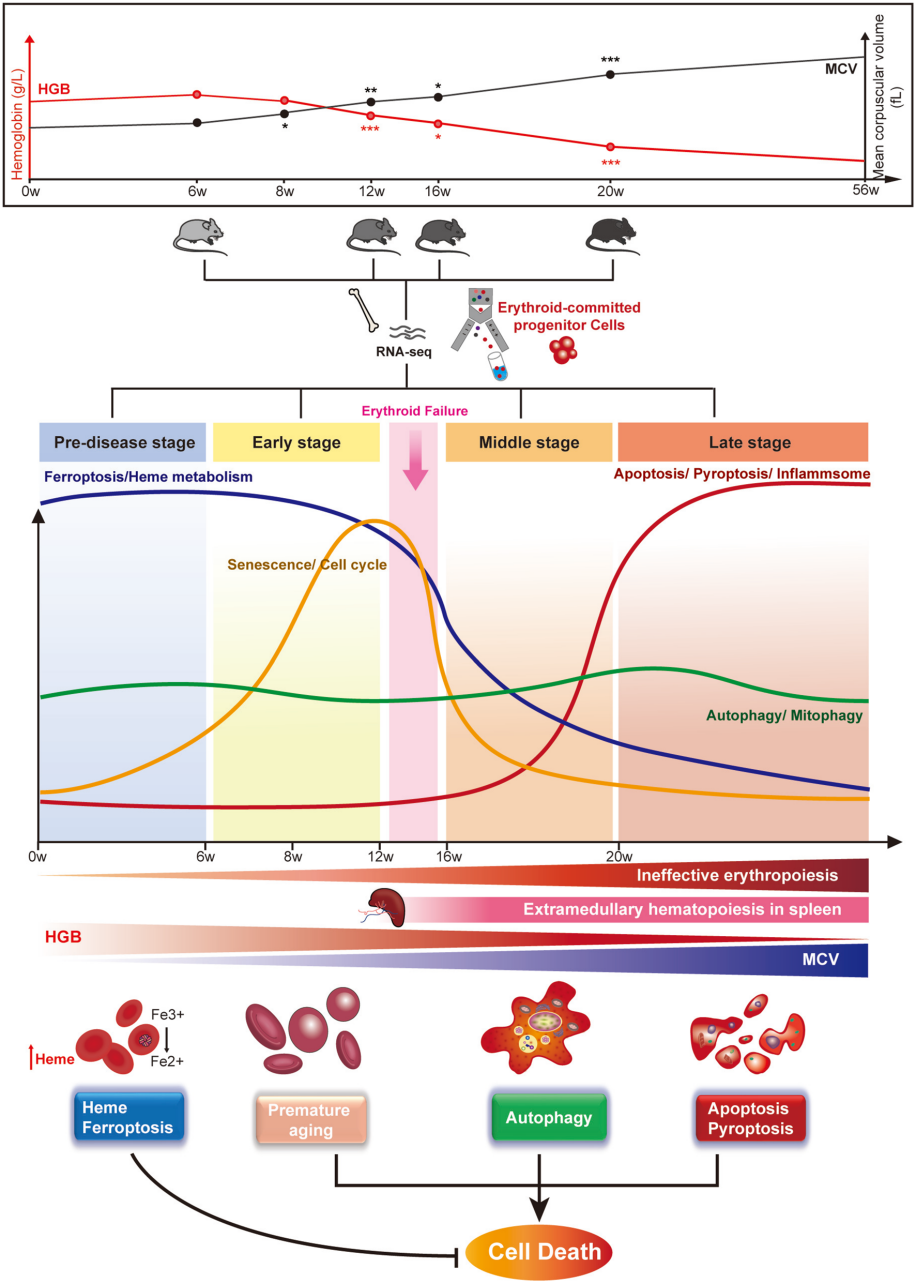
Dynamic signaling pathway alterations during progressive ineffective erythropoiesis in MDS. (1) The top panel shows changes in hemoglobin (HGB) and mean corpuscular volume (MCV) over time, with statistical significance (*P < 0.05, **P < 0.01, ***P < 0.001, ****P < 0.0001). (2) A timeline (0–56 weeks) tracks MDS progression, highlighting key processes such as ferroptosis, senescence, autophagy, and apoptosis. Erythroid progenitor cells from NHD13 and WT mice were sorted at selected four time point for RNA sequencing to identify gene expression changes. (3) The bottom panel depicts the molecular mechanisms leading to cell death, including dysregulated iron metabolism, premature aging, and activation of apoptosis and pyroptosis. Early MDS stages are driven by increased heme metabolism and ferroptosis, while later stages are characterized by senescence, cell cycle disruption, and inflammasome activation, indicating the potential for stage-specific therapeutic approaches in MDS

In conclusion, our study underscores the dynamic modulation of signaling pathways underlying ineffective erythropoiesis in MDS progression (Fig. 5). Preclinical and early stages are marked by upregulation of heme metabolism and ferroptosis pathways, progressing to the involvement of senescence and cell cycle pathways, and ultimately leading to the activation of apoptosis, pyroptosis, and inflammasome pathways. Dysregulated pathways at different stages of the disease suggest that stage-specific, targeted treatments may provide promising therapeutic strategies for MDS patients.

Our study has limitations. The profound anemia and diminished progenitor pool in NHD13 mice precluded functional validation through colony-forming assays. Although flow cytometry enables high-resolution isolation of immunophenotypically defined populations, these classifications may not fully recapitulate functional or developmental identities. Future investigations integrating single-cell transcriptomic and functional validation will be necessary to fully define erythroid hierarchies in pathological contexts.

## Supplementary Information

Additional file1

## Data Availability

The data that support the findings of this study are available on request from the corresponding author. The data are not publicly due to privacy or ethical restrictions.
