# Supplementary material for: A correlation of ineffective erythropoiesis and dysregulated signaling pathways in myelodysplastic syndromes/neoplasms
Source: Exp Hematol Oncol. 2025 May 14;14:71. doi: 10.1186/s40164-025-00664-1 (PMC12079896; doi:10.1186/s40164-025-00664-1)
Supplement: Supplementary file 1 — Additional file1 [file 40164_2025_664_MOESM1_ESM.docx]

**Supplementary Materials for “A correlation of ineffective erythropoiesis and dysregulated pathways in myelodysplastic syndromes/neoplasms”**

**Authors**

Junying Wu^1*^, Jinqin Liu^1*^, Jia Chen^1^, Fuhui Li^1^, Lin Yang^1^, Tiejun Qin^2^, Zefeng Xu^1,2^, Jing Liu^1^, Jiaxi Zhou^1^, Lihong Shi^1^, Bing Li^1,2^, Zhijian Xiao^1,2,3^

* These authors contributed equally to this study.

**Affiliation**

^1^State Key Laboratory of Experimental Hematology, National Clinical Research Center for Blood Diseases, Haihe Laboratory of Cell Ecosystem, Institute of Hematology and Blood Diseases Hospital, Chinese Academy of Medical Sciences & Peking Union Medical College, Tianjin, China.

^2^MDS and MPN Centre, Institute of Hematology and Blood Diseases Hospital, Chinese Academy of Medical Sciences & Peking Union Medical College, Tianjin, China.

^3^Hematologic Pathology Center, Institute of Hematology and Blood Diseases Hospital, Chinese Academy of Medical Sciences & Peking Union Medical College, Tianjin, China.

**Supplementary Methods**

**Patients**

A total of 1208 newly diagnosed primary MDS patients between August,2016 and June,2023 in our center were included in the study. Retrospective complete blood count (CBC) before diagnosis were collected from all patients, and 783 of them had traceable CBC information prior to diagnosis. Written informed consent was obtained from all patients in accordance with the Declaration of Helsinki. The study was approved by the Ethics Committee of the blood disease hospital, Chinese Academy of Medical Sciences & Peking Union Medical College.

**Mice**

C57BL/6 (WT) and C57BL/6-Tg (Vav1-NUP98/HOXD13) G2Apla/J (NHD13) mice^1^ were purchased from Jackson Laboratory and all mice were of a pure C57BL/6 genetic background. Based on the severity of anemia and changes in mean corpuscular volume (MCV), we selected four time points for NHD13 mice: 6 weeks (without anemia), 12 weeks (with mild anemia), 16 weeks (with obvious anemia), and 20 weeks (with severe macrocytic anemia). Age-matched wild-type mice were utilized in the experiments All animal studies were approved by the Institutional Animal Care and Use Committee of State Key Laboratory of Experimental Hematology. Mice were housed in the animal barrier facility at the State Key Laboratory of Experimental Hematology.

**Peripheral blood analyses**

Peripheral blood (PB) was collected from the retro-orbital vein of NHD13 and WT mice starting at 6 weeks of age and analyzing using an automatic blood cell analyzer (SYSMEX XN-1000). A drop of blood was collected from the retro-orbital vein of the mice and placed on a glass slide. Three to five smears were prepared. One slide was stained with Wright's stain and then examined under microscope.

**Immunohistochemical analysis**

Mouse tissue samples were fixed in 10% neutral-buffered formalin for 24h and embedded in paraffin. Paraffin blocks were sectioned at 3 μm and stained with hematoxylin and eosin (H&E). Hematoxylin-eosin (HE) staining procedure involved dewaxing and rehydrating the samples initially with xylene twice, followed by a xylene (1:1) solution, then twice with 100% ethanol, 80% ethanol, and finally distilled water. The samples were then stained with hematoxylin for five minutes. Subsequently, they were treated with 1% hydrochloric acid in ethanol for 30 seconds and rinsed with water. After staining with eosin for 1-3 minutes and washing with distilled water, the samples were dehydrated through a sequence of 80% ethanol, 95% ethanol twice, 100% ethanol twice, and finally xylene three times.

**Isolation and processing of** **murine bone marrow nucleated cells**

Murine bone marrow nucleated cells (BMNCs) were harvested by flushing the femur and tibia with PBS containing 2% Fetal Bovine Serum (FBS, Gibco) and 2 mM EDTA (Invitrogen). The cells were then lysed using red blood cell (RBC) lysis buffer (Solarbio) for 15 minutes and centrifuged at 4°C. Post-centrifugation, the cells were resuspended in PBS, filtered through a cell strainer, and subsequently counted.

**Flow cytometric analysis and sorting**

Murine BM cells were isolated following the method previously described. Spleen cells were processed by homogenizing the tissue and filtering through a 70-μm cell strainer (BD Biosciences). Peripheral blood (PB) was obtained from the retro-orbital vein. Single-cell suspensions from BM, spleen, and PB underwent staining with specific antibodies in a solution of 2% FBS/PBS for 30 minutes on ice after undergoing red blood cell (RBC) lysis, with the exception of erythroid precursors which were stained prior to RBC lysis. For surface staining, antibodies used included a Lineage Antibody Cocktail (17A2; RB6-8C5; RA3-6B2; Ter-119; M1/70, Biolegend), CD117 (c-kit, 2D8, BD Biosciences), Ly-6A/E (Sca-1, D7, BD Biosciences), CD34 (RAM34, invitrogen), CD16/CD32 (FcγRⅡ/Ⅲ, 93, invitrogen), CD71 (RI7217, Biolegend), CD41 (MWReg30, Biolegend), Ter-119 (Ter119, BD Biosciences), CD44 (QA19A43, Biolegend), Ly6C and Ly6G (Gr-1, RB6-8C5, Biolegend), CD11b (M1/70, BD Biosciences) and CD45 (30-F11, Biolegend). DAPI (Beyotime) was included to exclude dead cells prior to analysis.

Subsequent analyses were conducted on a FACS Canto Ⅱ flow cytometer (BD Biosciences) and data were processed using FlowJo software version 10 (Tree Star Inc.). The gating strategy of terminal erythropoiesis was followed as outlined by Liu *et al.*^2^. Erythroid differentiation within the samples was determined by quantifying the proportion of each terminal erythropoiesis stage in each BM sample. BM cells underwent sorting on a BD FACS Aria Ⅲ (BD Biosciences).

**RNA extraction and real-time quantitative PCR analysis**

Murine erythroid-committed progenitor (ErP, Lin^-^ckit^+^Sca-1^−^CD34^−^Fcγ^−^CD71^+^) cells were sorted using FACS Aria III flow cytometer (BD Biosciences). Total RNA from murine ErPs or human BMNCs was purified with Trizol (Invitrogen). For cDNA synthesis, 1 μg of total RNA was reverse transcribed using the RevertAid First Strand cDNA Synthesis Kit from Thermo Fisher Scientific. The RT-qPCR was then performed using the PowerUp^TM^ SYBR^TM^ Green Master Mix (Applied Biosystems) on a StepOne Real-Time PCR System (Thermo Fisher Scientific). All PCR assays were executed in triplicate to ensure accuracy. GAPDH was utilized as a reference gene to normalize the RNA content across samples. Specific primers were sourced from PrimerBank (https://pga.mgh.harvard.edu/primerbank/), as detailed in **Table S2**.

**Preparation and observation of transmission electron microscopy samples**

Murine bone marrow (BM) cells were isolated using a previously described method. Cell clumps were combined with pre-chilled serum in a 1.5 ml Eppendorf tube, homogenized, and centrifuged at 3000 rpm for 10 minutes to sediment the cells, with the supernatant discarded thereafter. The cell pellets were fixed in 2.5% glutaraldehyde at 4°C for 4 hours, washed four times with 0.1 mol/L phosphate-buffered saline (PBS), and then the serum-embedded samples were sectioned into roughly 2 mm^3^ blocks.

These samples were further fixed in 1% osmium tetroxide for 2 hours at 4°C, washed twice with PBS. Dehydration was carried out on a rocker for 45 minutes using graded acetone solutions (30%, 50%, 70%, 90%, and 100%), each for 15 minutes. Infiltration and embedding in resin followed, using acetone to resin ratios of 1:1 for 2 hours and 1:2 for 2 hours, then overnight in pure resin.

Polymerization was achieved by incubating the samples at 37°C for 12 hours, 45°C for 12 hours, and 60°C for 24 hours. The hardened resin blocks were then sectioned at 60-70 nm thickness with an ultramicrotome, stained with uranyl acetate and lead citrate, and analyzed using transmission electron microscopy. Images were taken after adjusting focus at various magnifications.

**References**

1. Lin YW, Slape C, Zhang Z, et al. NUP98-HOXD13 transgenic mice develop a highly penetrant, severe myelodysplastic syndrome that progresses to acute leukemia. *Blood*. 2005;106(1):287-95.

2. Liu J, Zhang J, Ginzburg Y, et al. Quantitative analysis of murine terminal erythroid differentiation in vivo: novel method to study normal and disordered erythropoiesis. *Blood*. 2013;121(8):e43-9.

**Supplementary Table 1.** Demographic, hematologic and clinical characteristics of 783 patients with primary MDS at diagnosis.

| **Demographic** | **No./****No.** **(%) (IQR)** |
| --- | --- |
| Patients, No. | 783 |
| Female/male, No. (%) | 291 (37.1)/493 (62.9) |
| Age, years (range) | 56 (45-64) |

| **2016 WHO Category** | **No. (%)** | **2022 WHO Category** | **No. (%)** | **2022 ICC Category** | **No. (%)** |
| --- | --- | --- | --- | --- | --- |
| MDS-5q- | 13 (1.7) | MDS-LB-5q- | 12 (1.5) | MDS-*SF3B1* | 60 (7.7) |
| MDS-SLD | 69 (8.8) | MDS-LB-*SF3B1* | 78 (10.0) | MDS-del(5q) | 13 (1.7) |
| MDS-MLD | 310 (39.6) | MDS-bi*TP53* | 54 (6.9) | MDS, NOS without dysplasia | 2 (0.3) |
| MDS-RS-SLD | 31 (4.0) | MDS-LB | 296 (37.8) | MDS, NOS, with SLD | 75 (9.6) |
| MDS-RS-MLD | 49 (6.3) | MDS-h | 75 (9.6) | MDS, NOS, with MLD | 309 (39.5) |
| MDS-EB1 | 151 (19.3) | MDS-IB1 | 121 (15.5) | MDS-EB | 151 (19.3) |
| MDS-EB2 | 137 (17.5) | MDS-IB2 | 95 (12.1) | MDS/AML | 102 (13.0) |
| MDS-U | 23 (2.9) | MDS-f | 25 (3.2) | MDS with mutated *TP53* | 33 (4.2) |
|  |  | CCUS | 6 (0.8) | MDS/AML with mutated *TP53* | 18 (2.3) |
|  |  | AML | 21 (2.7) | CCUS | 7 (0.9) |
|  |  |  |  | AML | 13 (1.7) |

| **Hematologic Feature** | **Median (IQR)** |
| --- | --- |
| Hemoglobin, g/dL | 79 (65-95) |
| Neutrophils, ×10^9^/L | 1.11 (0.66-1.95) |
| Platelets, ×10^9^/L | 66 (34-137) |
| Mean corpuscular volume, fL | 100 (92.9-107.7) |

| **Clinical Feature** | **No. (%)** |
| --- | --- |
| Cytogenetic risk according to IPSS-R criteria, n=637 |  |
| Very good | 7 (1.1) |
| Good | 356 (55.9) |
| Intermediate | 166 (26.1) |
| Poor | 38 (6.0) |
| Very poor | 70 (11.0) |
| IPSS-R risk group No, n=691 |  |
| Very low | 33 (4.8) |
| Low | 176 (25.5) |
| Intermediate | 238 (34.4) |
| High | 137 (19.8) |
| Very high | 107 (15.5) |
| IPSS-M risk group No, n=701 |  |
| Very low | 19 (2.7) |
| Low | 144 (20.5) |
| Moderate low | 109 (15.5) |
| Moderate high | 108 (15.4) |
| High | 161 (23.0) |
| Very high | 160 (22.8) |

Abbreviations: IQR: inter-quartile ranges; WHO, World Health Organization; MDS: myelodysplastic syndromes(neoplasms); 5q-: isolated 5q deletion; SLD: single lineage dysplasia; MLD: multilineage dysplasia; RS-SLD: ring sideroblasts with SLD; RS-MLD: ring sideroblasts with MLD; EB1/2: excess blasts type 1/2; MDS-U: MDS unclassifiable; LB: low blasts; MDS-LB-*SF3B1*: MDS with low blasts and *SF3B1* mutation; bi*TP53*: biallelic *TP53* inactivation; MDS-h: MDS, hypoplastic; IB1/2: increased blasts type1/2; MDS-f: MDS with fibrosis; CCUS: clonal cytopenia of undetermined significance; AML: acute myeloid leukemia; MDS-*SF3B1*: myelodysplastic syndromes with mutated *SF3B1*; MDS, NOS: myelodysplastic syndromes, not otherwise specified; MDS/AML: myelodysplastic syndrome/ acute myeloid leukemia; IPSS-R: International Prognostic Scoring System-Revised; IPSS-M: International Prognostic Scoring System-Molecular.

**Supplementary Table 2.** List of primers for qPCR.

| **Gene** | **Forward (sense)** | **Reverse (antisense)** |
| --- | --- | --- |
| *mGpx4* | TGTGCATCCCGCGATGATT | CCCTGTACTTATCCAGGCAGA |
| *mNcoa4* | CCTGGGGCAATCTGAAGGG | CTGAGGAGTCACCAACCAATC |
| *mAcsl6* | GAACTCAACTACTGGACCTGC | CCGTGGACGTAGATTTGTGC |
| *hNCOA4* | ACAGTTGCATAAGCCGTCACC | TGAGCCTGCTGTTGAAGTGTC |

**Supplementary Table 3.** Causes of Mortality in NHD13 Mice.

| **Cause of Mortality** | **Percentage of Mice Affected** | **Observations** |
| --- | --- | --- |
| Anemia | 60% | low RBC count and pale appearance |
| Acute myeloid leukemia | 20% | peripheral blood blasts or bone marrow blasts >20% |
| Infections | 10% | respiratory distress, hepatosplenomegaly, elevated neutrophils |
| Multi-organ failure | 5% | general body swelling, organ degeneration |
| Thrombosis | 5% | thrombosis in the intestines, lungs and spleen |

**Supplemental Figure 1. Flow cytometric analysis of erythroid progenitor populations in mouse bone marrow.** Representative flow cytometric images of CFU-E and pre CFU-E among live BM cells and erythroid progenitor (ErP) populations in WT (A) and NHD13 mice (B). (C) Flow cytometry analysis of CFU-E and pre-CFU-E subsets within the bone marrow ErP population at 4 time points in WT and NHD13 mice (6, 12, 16, and 20 weeks). Data are presented as mean ± SEM; ns, not significant (unpaired two-tailed t-test).

**Supplemental Figure 2.** **Flow cytometric analysis and representative cytospin images of the sorted populations of age-matched wild-type (WT) and NHD13 mice bone marrow cells.** (A-F) Flow cytometric analysis and isolation of erythroblasts of WT mice bone marrow cells. (G) Representative cytospin images of the sorted populations in WT mice. (H-M) Flow cytometric analysis and isolation of erythroblasts of NHD13 mice bone marrow cells. (N) Representative cytospin images of the sorted populations in NHD13 mice. All cellular proportions in the image represent percentages relative to viable cells. Ⅰ: proerythroblasts; Ⅱ: basophilic erythroblasts; Ⅲ:polychromatic erythroblasts; Ⅳ: orthochromatic erythroblasts; Ⅴ:reticulocytes; Ⅵ: mature red blood cells. Scale bar, 50μm.


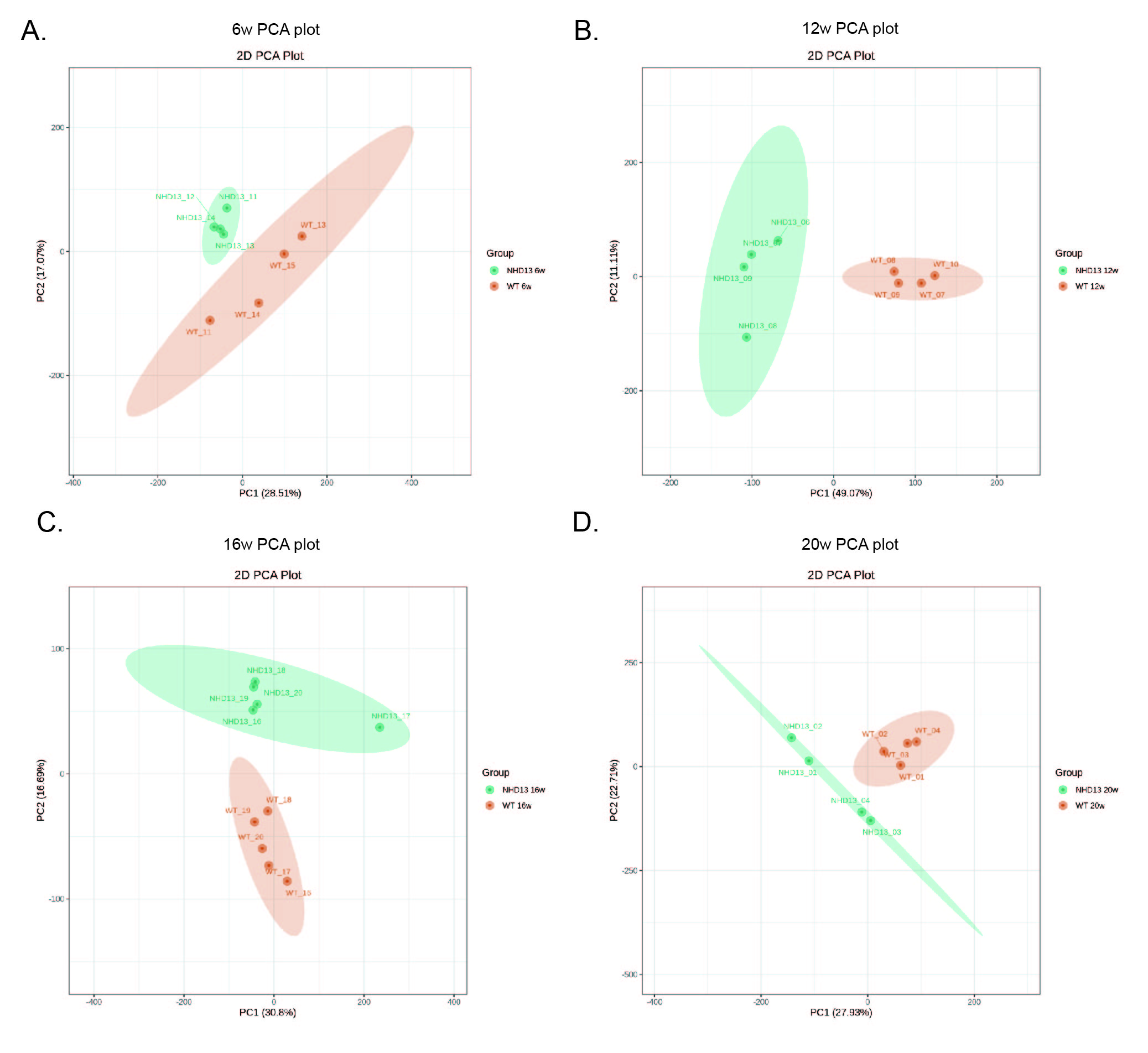


**Supplemental Figure 3. Principal component analysis (PCA) of RNA sequencing data from WT and NHD13 mice at different time points**. (A) PCA plot of the RNA sequencing data at 6 weeks. (B) PCA plot of the RNA sequencing data at 12 weeks. (C) PCA plot of the RNA sequencing data at 16 weeks. (D) PCA plot of the RNA sequencing data at 20 weeks.


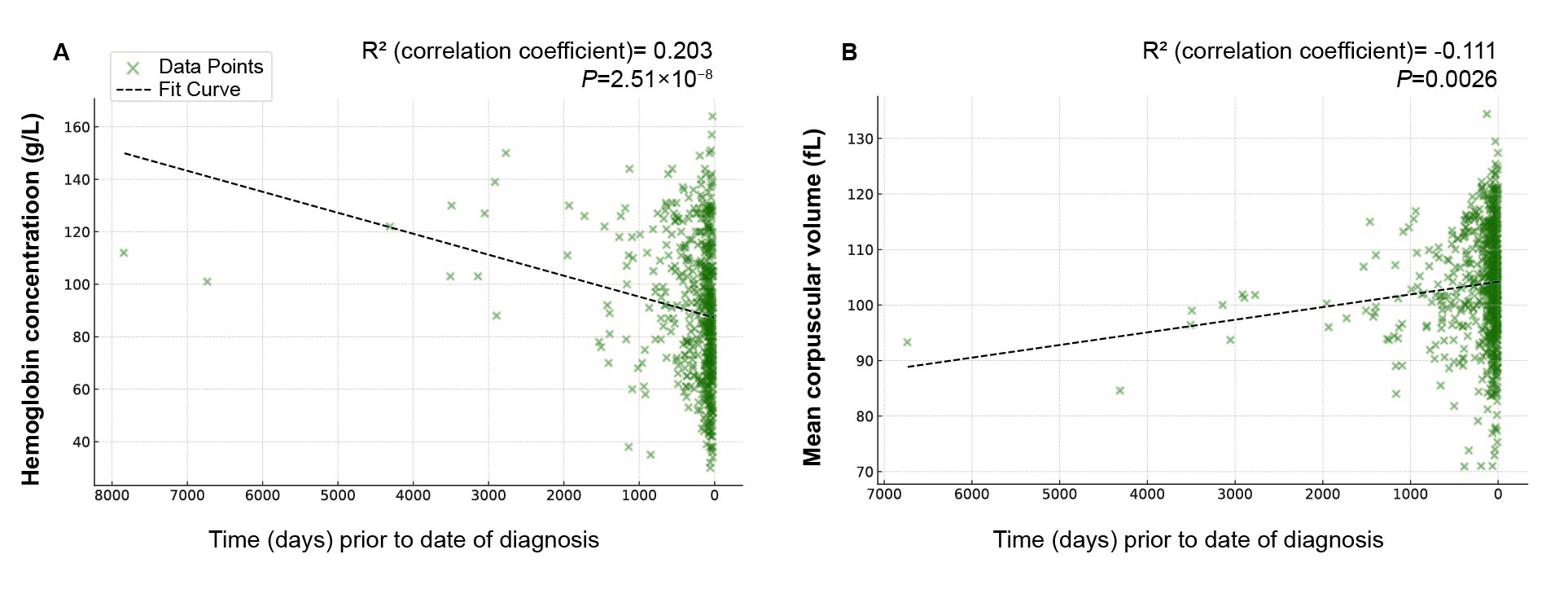


**Supplemental Figure 4. Hemoglobin (HGB) levels and mean corpuscular volume (MCV) changes in primary MDS patients prior to date of diagnosis**. (A) Changes in HGB levels before diagnosis. (B) Changes in MCV before diagnosis. The black dashed line represents the best fit curve.


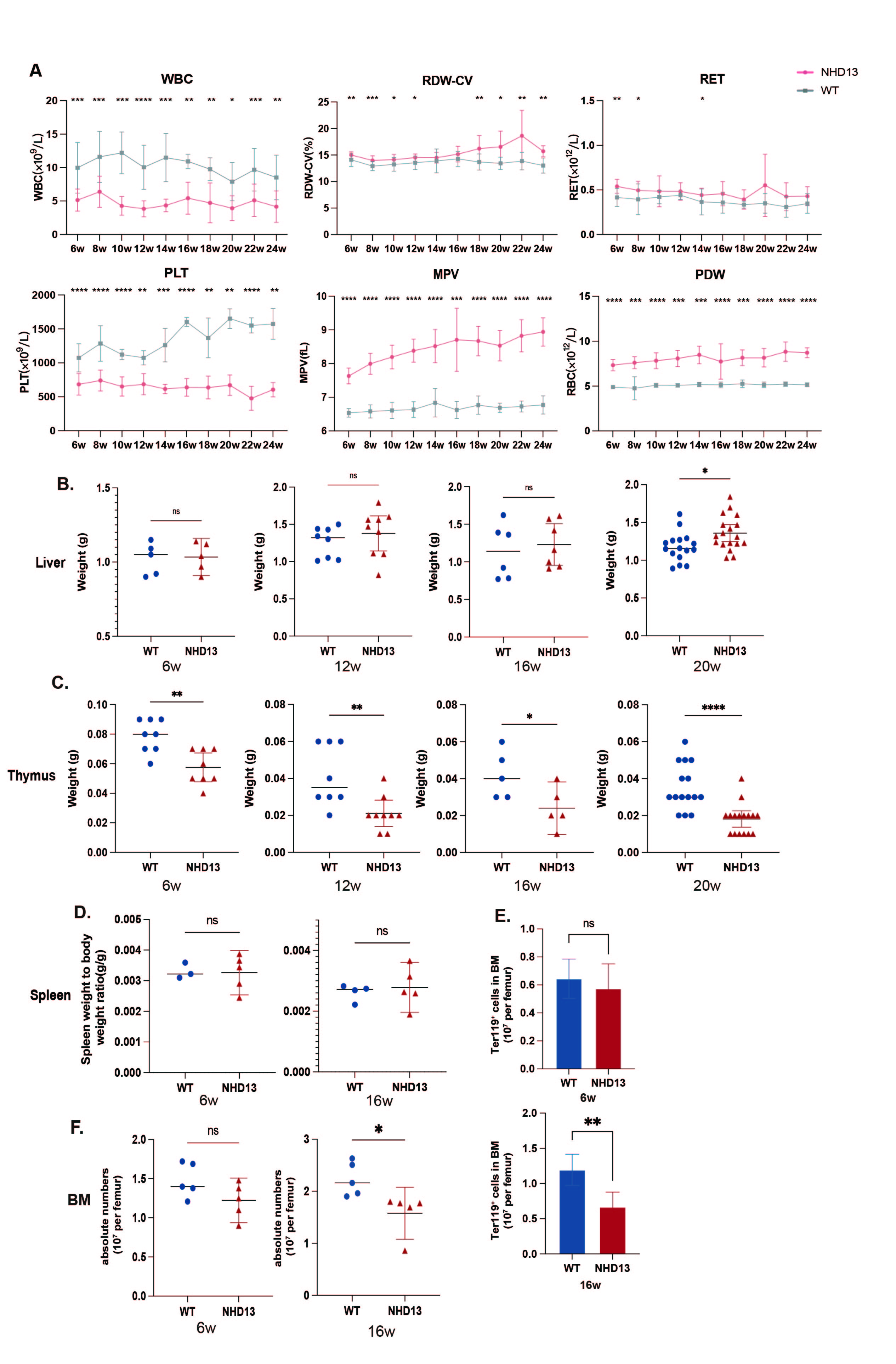


**Supplemental Figure 5. Disease phenotype changes in NHD13 and WT mice at different ages.** (A) Peripheral blood (PB) analyses of NHD13 and WT mice at various time points, showing progressive changes in hematological parameters (n = 12–20 mice per group).
(B-C) Comparison of liver (B) and thymus (C) weights between NHD13 and WT mice at different ages. (n=6-15 mice/group). (D) Spleen weight relative to body weight between NHD13 and WT mice at 6 and 16 weeks (n = 3-5 mice per group). (E) absolute Ter119^+^ erythroid cells per femur at 6 and 16 weeks of age (n = 5 mice per group). (F) Absolute BM cell numbers per single femur at 6 and 16 weeks of age (n = 5 mice per group). WBC: white blood cell; RDW-CV: red blood cell distribution width; RET: reticulocyte; PLT: platelet; MPV: mean platelet volume; PDW: platelet distribution width. **P*<0.05, ***P*<0.01, ****P*<0.001, *****P*<0.0001.


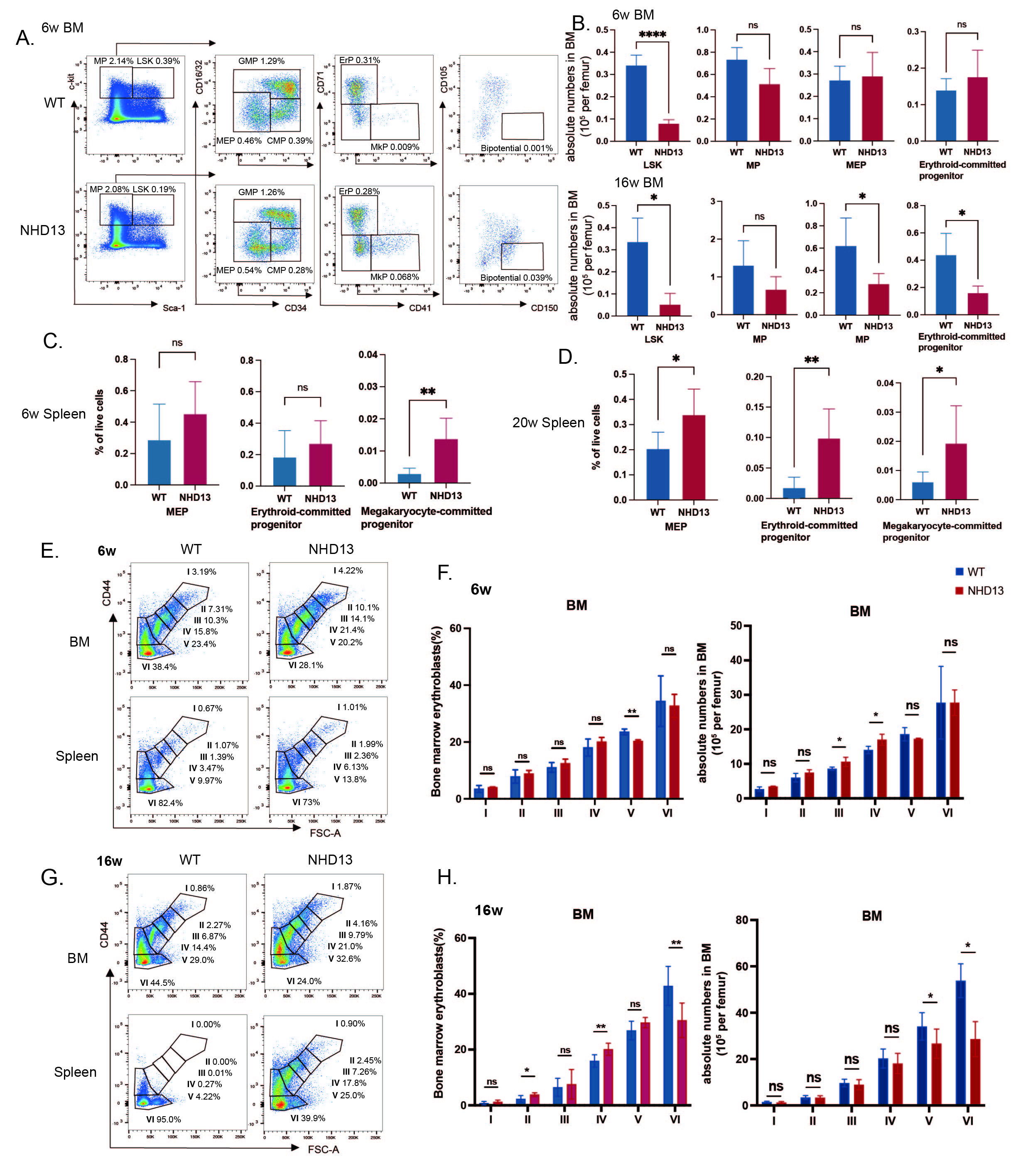


**Supplemental Figure 6. Erythroid progenitor cell exhaustion and impaired terminal erythropoiesis are observed in NHD13 mice.** (A-B) Representative flow cytometric plots (A) and quantification of hematopoietic cell populations (B) in the bone marrow (BM) of NHD13 and WT mice at 6 and 16 weeks of age (n = 5-6 per group). Absolute cell numbers were normalized to a single femur. (C-D) Analysis of hematopoietic cell populations in the spleens of NHD13 and WT mice at 6 weeks (C) and 20 weeks (D) of age. (E-H) Comparison of the proportions and absolute cell counts (per femur) of erythroblasts at different stages of terminal erythroid differentiation in the BM of NHD13 and WT mice at 6 (E-F) and 16 weeks (G-H) of age. CD44 levels and forward scatter (FSC) were analysed to define various developmental stages of erythroblasts (n=8-9 per group). The results are presented as mean ± standard error of the mean. A two-tailed unpaired Student t test was performed between means of two groups. ns, not significant, **P*<0.05, ***P*<0.01, ****P*<0.001, *****P*<0.0001. LSK: Lin^-^Sca-1^+^c-kit^+^ cells; MP: multipotent progenitors; GMP: granulocyte/macrophage progenitors; CMP: common myeloid progenitors; MEP: megakyocyte-erythroid progenitors; ErP: erythroid-committed progenitor (Lin^-^ckit^+^Sca-1^−^CD34^−^Fcγ^−^CD71^+^); MkP: Megakaryocyte-committed progenitors; Bipotential: bipotential progenitors.Ⅰ: proerythroblasts; Ⅱ: basophilic erythroblasts; Ⅲ:polychromatic erythroblasts; Ⅳ: orthochromatic erythroblasts; Ⅴ:reticulocytes; Ⅵ: mature red blood cells.


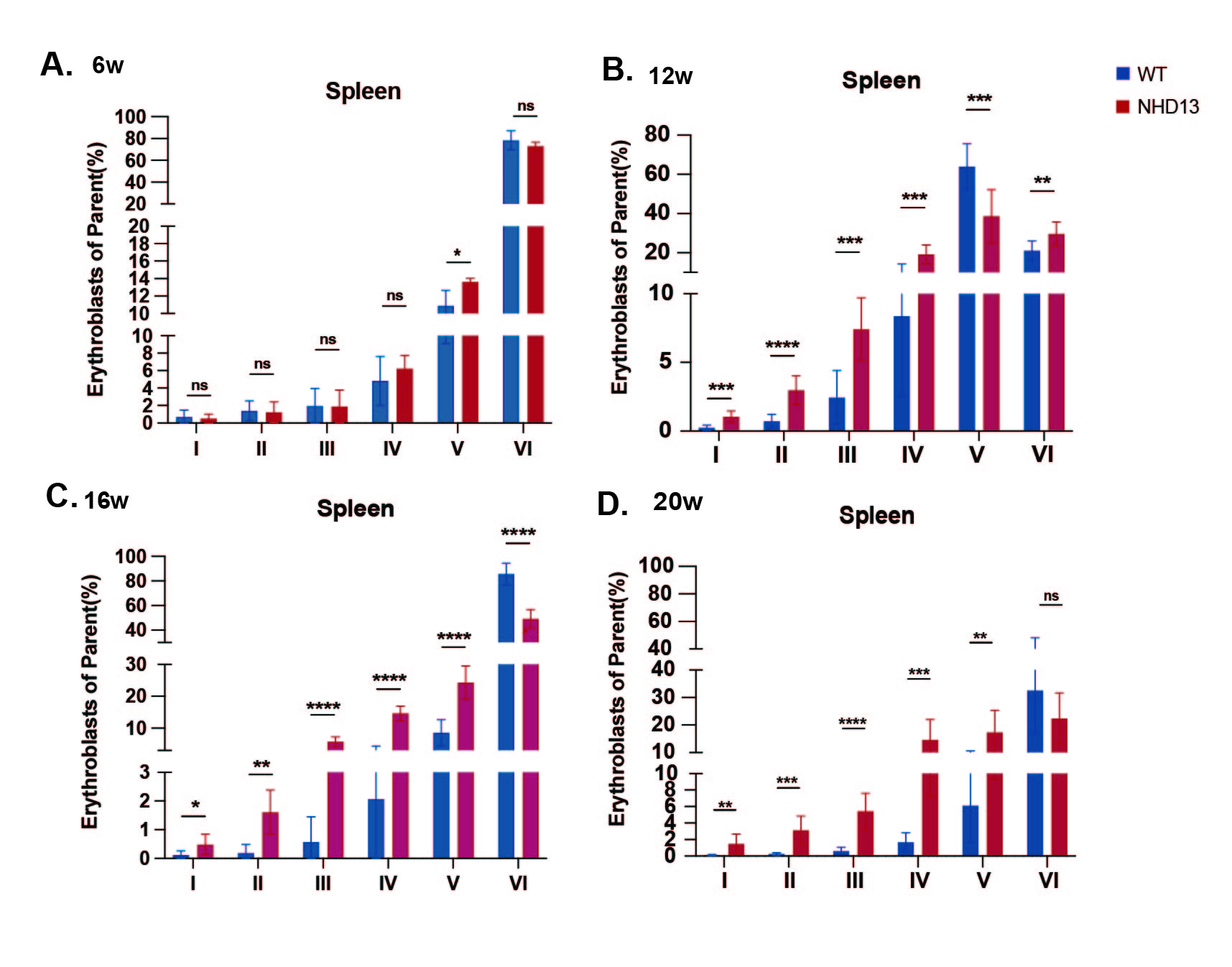


**Supplemental Figure 7. Erythroblast composition in the spleen of NHD13 and WT mice at different ages.** (A-D) Comparison of the proportions of erythroblasts at various stages of terminal erythroid differentiation in the spleen of NHD13 and WT mice at (A) 6 weeks, (B) 12 weeks, (C) 16 weeks, and (D) 20 weeks of age (n=8-9 per group). Erythroblast subsets were defined based on CD44 expression and forward scatter (FSC) profiles. Data are presented as mean ± SEM. A two-tailed unpaired Student t test was performed between means of two groups. ns, not significant, **P*<0.05, ***P*<0.01, ****P*<0.001, *****P*<0.0001.Ⅰ: proerythroblasts; Ⅱ: basophilic erythroblasts; Ⅲ:polychromatic erythroblasts; Ⅳ: orthochromatic erythroblasts; Ⅴ:reticulocytes; Ⅵ: mature red blood cells.


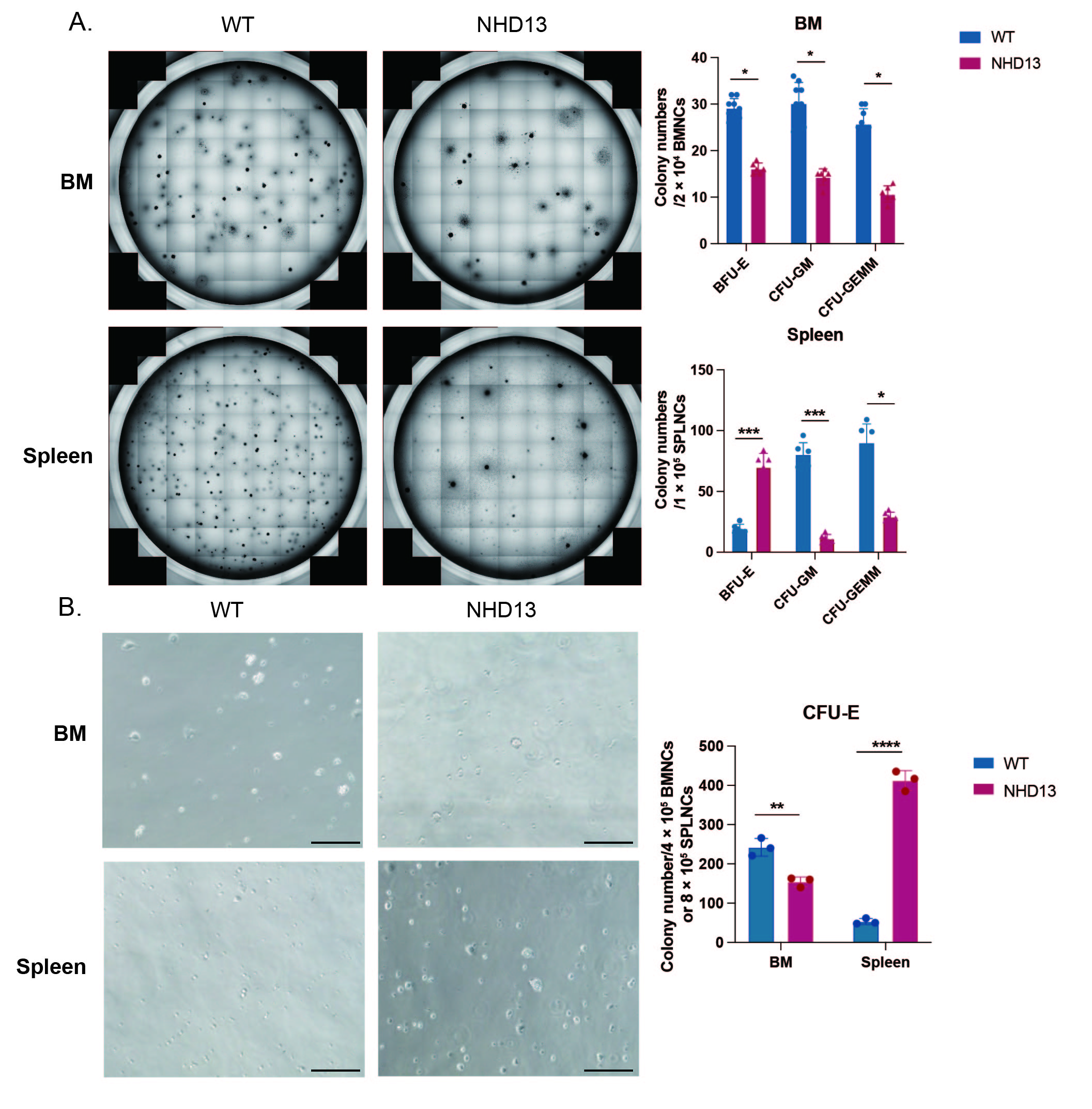


**Supplemental Figure 8. Comparison of colony-forming capabilities in vitro between severely anemic NHD13 mice and age-matched WT mice.** (A) Colony-forming assay showing the number of colonies formed after 7 days of ex vivo culture of 2 × 10⁴ bone marrow nucleated cells (BMNCs) and 1 × 10⁵ spleen nucleated cells (SPLNCs) from 20-week-old NHD13 and WT mice. Colony numbers are quantified for BFU-E, CFU-GM and CFU-GEMM.

(B) Count of CFU-E (Colony-Forming Unit-Erythroid) after 48 hours of culture from 4 × 10⁴ BMNCs and 8 × 10⁴ SPLNCs of NHD13 and WT mice. BFU-E: Burst forming unit-erythroid; CFU-GM: CFU-granulocyte, macrophage; CFU-GEMM: CFU-granulocyte, erythrocyte, macrophage, megakaryocyte; CFU-E: CFU-erythroid. Objective magnification 20×, scale bar 100μm. **P*<0.05; ***P*<0.01; ****P*<0.001; *****P*<0.0001.


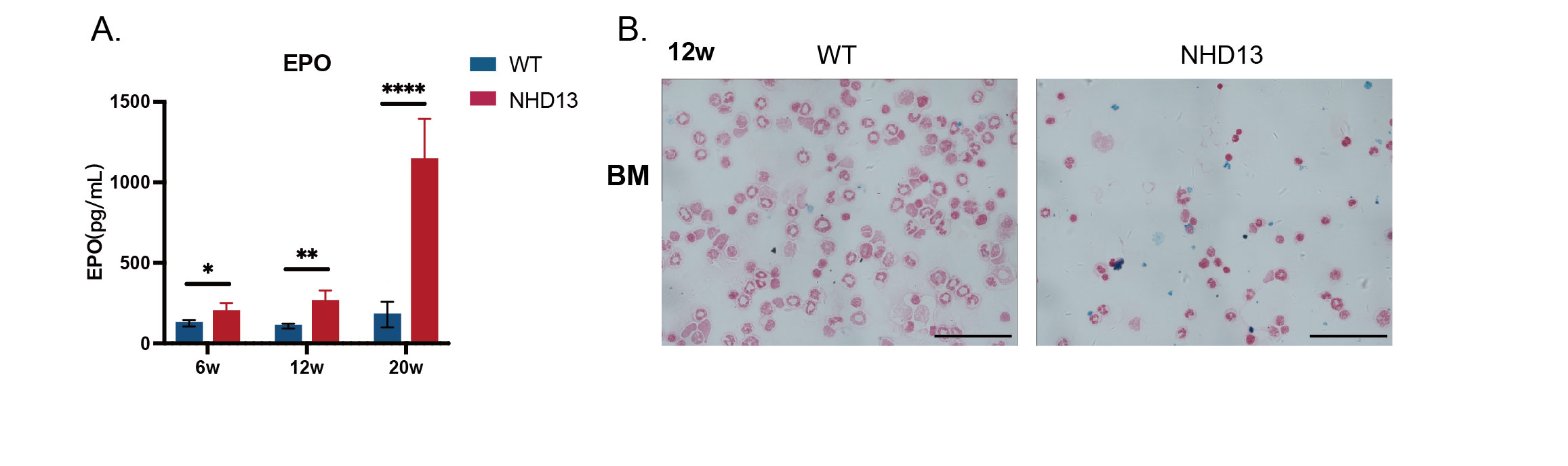


**Supplemental Figure 9.** **Iron-related characteristics of NHD13 mice in the early stage of MDS.** (A) NHD13 mice exhibit elevated EPO levels since preclinical stage of the disease (n=10-12 per group). (B) Prussian blue iron staining of cytospin bone marrow smears from NHD13 and WT mice showed an increase in both intracellular and extracellular iron in the BM cells at 12 weeks. Objective magnification 20×, scale bar 50μm. **P*<0.05; ***P*<0.01; ****P*<0.001; *****P*<0.0001.


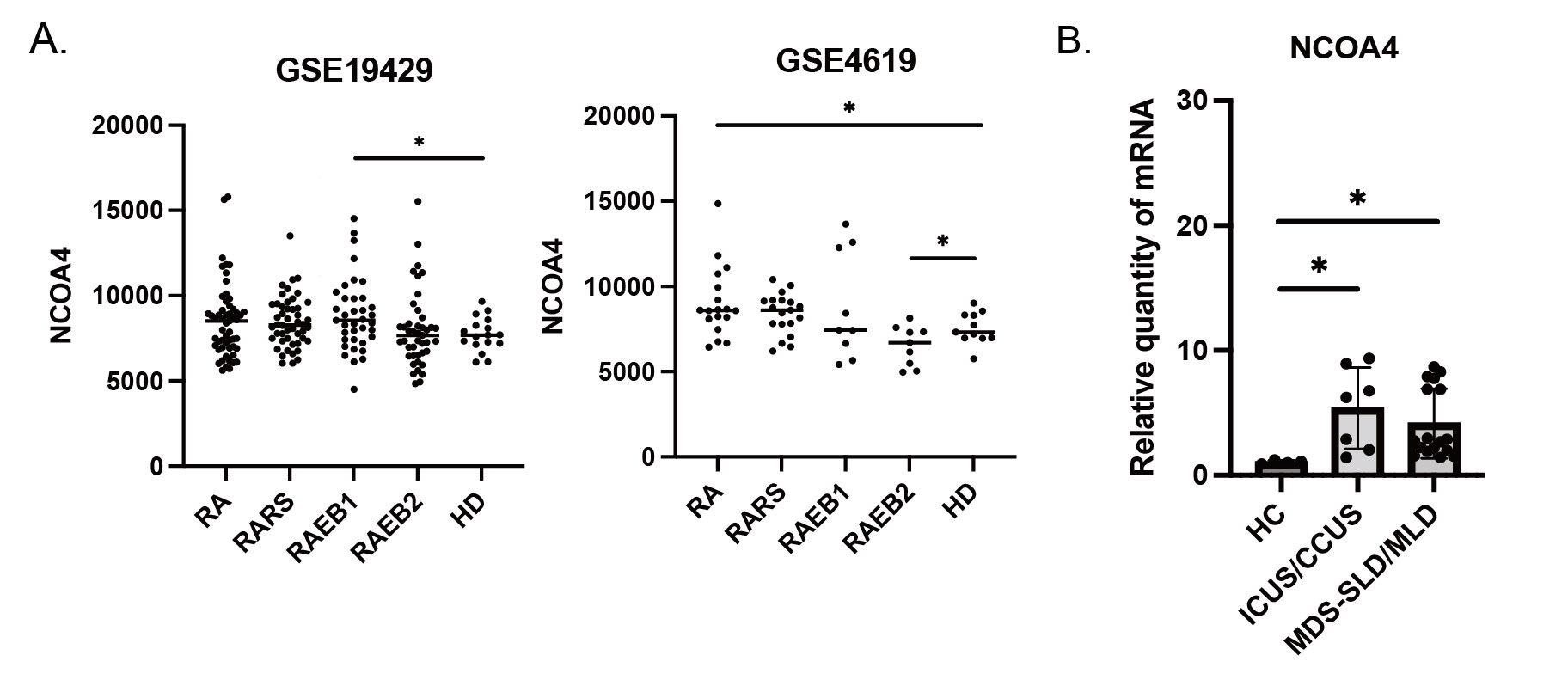


**Supplemental Figure 10. Differential expression of NCOA4 in MDS patients and healthy controls.** (A) Elevated NCOA4 gene expression in primary BM CD34^+^ cells from MDS patients versus healthy controls, analyzed using gene expression microarray data from GEO datasets (GSE19429 and GSE4619). (B) Quantitative PCR analysis showing higher NCOA4 expression in BM mononuclear cells from patients with ICUS, CCUS and lower-risk MDS (MDS-SLD and MDS-MLD) compared to healthy controls. ICUS: idiopathic cytopenia of undetermined significance; CCUS: clonal cytopenia of undetermined significance; SLD single lineage dysplasia; MLD: multilineage dysplasia (n=4-16 per group).
